# A survey on psychiatry trainees’ experiences of racism

**DOI:** 10.1192/bjo.2021.426

**Published:** 2021-06-18

**Authors:** Aicha Rais, Richard Burton, Adeel Rauf

**Affiliations:** Derbyshire Healthcare NHS Foundation Trust

## Abstract

**Aims:**

To measure rates of racism experienced and witnessed by trainees training in Derbyshire.

**Background:**

Derbyshire Healthcare Psychiatry trainee workforce comprises 39.1% white, 52.2% ‘Black, Asian and Minority Ethnic’ (BAME) and 8.7% undisclosed ethnicity. Racism can affect trainees by increasing risk of poor mental health and psychological distress leading to worse health outcomes. Discrimination, marginalisation and segregation is related to poorer quality education and employment opportunities.

**Method:**

Electronic surveys were sent out via e-mail to trainees in the North and South Derbyshire Hospital sites, accessible online from 11th to 31st December 2020.

Questions asked about personal experiences of racism, witnessing racism from patients and/or staff whilst training in Derbyshire. Trainees were asked if they know how to report incidents and if routinely reported. Trainee ethnicity was recorded.

**Result:**

A total of 56 trainees received a survey request. Response uptake rate 25% (14 out of 56). Respondents comprised of 36% white and 64% BAME doctors. Over one third (36%) of trainees reported experiencing racism from staff. 64% of trainees reported experience of racism from patients. There was no report of racism witnessed by staff towards patients. 29% of trainees reported witnessing both staff on staff racism and patient on staff racism. 93% of trainees reported witnessing racism from patients to staff. 29% BAME trainees reported experiencing racism from both staff and patients. 7% BAME trainees said they experienced racism from staff alone. 36% of trainees reported experiencing racism from patients only (4 BAME and 1 white trainee). 57% of trainees do not know how to report racism. 50% of trainees said they would report racism if they knew how.

**Conclusion:**

Racism remains a barrier affecting the lives of trainees requiring attention. BAME trainees are disproportionately affected by racism, and report witnessing more incidents, from staff and patients in the workplace. There remains an apprehension by doctors to report racism. A departmental presentation has been delivered on racism, unconscious bias, incident reporting process and sources of support. A workshop with the ‘Equality Diversity Inclusion’ team has been delivered to all trainees with the presence of the Freedom to Speak Up Guardian. Our British Medical Association Local Negotiating Committee Representative has also been informed.

